# The British Medical Association

**Published:** 1863-10

**Authors:** 


### Art. II.—THE BRITISH MEDICAL ASSOCIATION.

The recent meeting of the British Medical Association at Clifton is suggestive of many considerations. Perhaps at no period has it been more important than at the present that members of the medical profession should confidently and cordially co-operate for their mutual benefit, as well as for the interest of that science to which they are devoted. A very great change has of late years been effected in the professional and social status of medical men. They now occupy a much higher position in the opinion of the executive than heretofore, and by the public are recognised as authorities on those matters of hygienic economics which have hitherto been under the unquestioned control of the municipal powers. The medical profession, notwithstanding its progress, has a continuing necessity for the exercise of a combined discretion. Schism within and dangers without have invested the practice of medicine and surgery with no inconsiderable risks.

Medical associations bring together into friendly relationship men of similar tastes and kindred pursuits, and so establish a reciprocity of good feeling. They also enable abstruse questions to be investigated by fresh judgments without bias, according to the teaching of varied experiences, and thereby submit scientific problems to the most impartial and, at the same time,

---

severe tribunal, before which they can be discussed. They revive the interest in matters of the past, and give additional confidence for the future. A resolution of Dr. Radclyffe Hall in reference to the legal persecutions of Mr. Adams, Dr. Waters, and Dr. Philbrick, illustrates our first proposition. This resolution met with hearty approval. It afforded an opportunity "of deprecating the conduct of any members of the medical profession who render assistance in the legal persecution of their brethren on mere suspicion, or on grounds which have not even the semblance of being substantial." This is a step in the right direction, and promises to lead to the termination of such attempts as we have alluded to, wherein the courage of those accused happily prevailed. Can it be doubted that cases do exist in which, rather than "be dragged through such a trial," compromises have been effected on the behalf of blameless men, who by such weakness have supplied the means of their lifelong extortion and misery? Outside the medical profession analogous examples are not wanting. Within the medical profession false charges of such a nature could not hope for success, if medical men were only true to each other. This reiteration of professional sentiment will further contribute to limit the opinions of medical men on medico-legal questions to their scientific conclusions on distinct propositions embodying facts, not as has been complained of in such cases, their personal and general estimate of pronounced propositions assuming facts. Such limitation will do much to exalt the character of medical men as expert witnesses. This resolution of the Association is, therefore, fraught with good service to the profession at large.

The President, Dr. Symonds, delivered an able address, in which he inquired "How has the art of medicine advanced in the opinion of the public?" This is now more easily propounded than demonstrated. We may safely assume that the medical profession *has* advanced, and never before occupied a higher or prouder position than it does at present. The commendable prudence suggested by the American's remark, "your conclusions may be right, but your reasons may be wrong," advise us not to investigate too minutely the processes by which such has been accomplished. And yet we desire not to beg the question. We affirm that every advance in the social status of the medical profession is due to the profession itself and its operation upon the public judgment. Disciplined professional opinion, guiding the medical press, while advising those in practice, has, at the same time, impressed the public with principles which, though previously acknowledged, have not hitherto been permitted to assume demonstrative reality. Medical men are now recognised as public officers, entitled to a distinct status and to public support. In the military and naval services their rewards are increased and their rank permitted to progress to that degree which affords them position and pay, as far as is consistent with the influence of command. In the several governmental departments increased, and in many instances liberal, remuneration is awarded them. In their relationship with public institutions an independence is conceded which constitutes them, in the performance of their duties, masters of their positions. Their several colleges are brought into distinct association with the state through the general council, and each member of the profession has therein the means of insuring protection or demanding redress. These advances have been imperceptibly made. These advantages the medical profession has achieved for itself by the steady co-operation of earnest men, and by the force of professional example influencing the public mind. Much is due to associations such as that we comment on. Much is due to the hearty co-operation of medical men whenever a professional wrong is to be redressed, or professional right to be vindicated. *Divide et Impera* is a motto medical communities in provincial towns would therefore do well to bear in mind, for most certainly it is to local disagreements that we have traced almost every instance of alleged professional oppression that it has been our duty to comment on.

While congratulating the profession on that which it has deservedly achieved, we must not lose sight of the fact, that many sources of grave professional anxiety exist. In confirmation of our second proposition, that such associations lead to the free discussion of scientific questions, we need scarcely, with the "proceedings" of the late meeting before us, offer arguments. We prefer to investigate the sources of anxiety to which we allude, and these we find, while involving scientific considerations of the first importance, to yet rest in causes not immediately under professional control. A large amount of medical scepticism exists. This manifests itself either in the distrust of legitimate medicine and the employment of specifics, or the repudiation of medicine and the adoption of special systems. Empiricism in the one and scepticism in the other instance, both eventuate in the practical abandonment of those principles by which medicine as a science reduces its observation and teaching to practice, giving to theory an eclectic reality, which for the treatment of disease and the purposes of life is available as an art. This condition of mind on the part of the public, and the successful sale of such remedies, are chiefly due to the active habit of inquiry characteristic of our age, as well as to the adventurous spirit of speculation which clothes the wildest schemes in specious promises, and offers hopes to the most desperate ambition. Did the use of specifics merely lead to the transferring the payments of the ignorant and credulous to the pockets of those unprincipled pretenders who unblushingly proclaim their capabilities of treating every disease by a kind of universal cure, the mischief resulting therefrom would be by no means inconsiderable. Unfortunately the evil takes a wider range. Specialists and specifics too frequently go together, and pander to the vicious while they plunder the foolish. Such practitioners are generally outside the pale of the profession, and it is well that in every assemblage of honourable medical men their conduct be fittingly commented on, as it was by the profession assembled at Clifton. The Medical Council have already intimated their intention of advising steps for the diminution if not complete prevention of this evil. It is to be hoped that no sentimentalism on the part of the executive will prevent their propositions receiving practical effect. The injury which such a class inflicts on the medical profession is slight in comparison with that which results from the want of medical truth, which has induced many of its recognised members to adopt strange theories and pursue practices in direct variance to that teaching and study by which they have attained their qualifications. That many men of genius and learning advocate and adopt a line of practice inconsistent with legitimate medicine is a matter of common knowledge. That the public, to a large extent, countenance homœopaths, mesmerists, and others who traffic on their weakness when combined with their illness, is undoubtedly true. That the arguments urged as against medicine are not for one moment tenable will be at once established by a brief survey of the reasons in their support most generally advanced. It is urged that medicine as a science is not reliable, for that its practice is mutable. True—the nature of disease changes, and the course of remedial action must proportionally vary. It is a narrow philosophy which presumes that when all around is undergoing alteration, temperature, soil, habits, associations, and diet, that the human constitution, on the one hand, and the elements influencing it on the other, are not proportionally affected; even if the character of disease had not something beyond, and independent of both, to establish its type. Arguments founded on difference of treatment for alleged identical diseases, instead of being adverse to the reliable character of medicine, constitute so many inferences in its favour as a progressive science capable of adaptation to the varying exigencies of each separate case. It is further declared that a recognised pharmacopœia is but the instrument of disciplined empiricism which prescribes remedies in ignorance of their *modus operandi*, and is obliged to retrace their action to learn their properties.

True it is so: but how closely is that empiricism identified with science, which confers the power not only of diagnosis but of predication! which tells what is, and foresees what will follow by the application of remedies, which recognises that which exists and also enables man to anticipate changes in the living laboratory of the human body as the result of the inexplicable influence exercised by remedies administered in the full confidence of medical faith. Again, it is urged that a science cannot rest on firm foundations which permits equally competent votaries to arrive at widely different conclusions on the same question. This is the least tenable argument that can be advanced. Inasmuch as in the exact sciences, where demonstration and material substances are under consideration, men of the largest experience arrive at different conclusions without the science they advocate being thereby impeached. Not the least of the many advantages which must result from the assembling together of members of the medical profession is their practical refutation of such impressions, which ignorance suggests, and prejudice or self-interest too frequently maintains.

There can be no doubt that such associations and meetings of medical men for the investigation of scientific questions, must be attended with happy results, whether it be by the drawing into closer bonds of fellowship and bringing into more intimate relations practitioners who otherwise might not become personally acquainted, or whether it be by the free discussion of questions of general or of abstract science, it matters not, since the practical end accomplished must be the promotion of the welfare, and the advancing the objects, of a profession in which we each feel a common interest and experience a common pride.

